# The impact of COVID-19 pandemic on physical and mental health of Asians: A study of seven middle-income countries in Asia

**DOI:** 10.1371/journal.pone.0246824

**Published:** 2021-02-11

**Authors:** Cuiyan Wang, Michael Tee, Ashley Edward Roy, Mohammad A. Fardin, Wandee Srichokchatchawan, Hina A. Habib, Bach X. Tran, Shahzad Hussain, Men T. Hoang, Xuan T. Le, Wenfang Ma, Hai Q. Pham, Mahmoud Shirazi, Nutta Taneepanichskul, Yilin Tan, Cherica Tee, Linkang Xu, Ziqi Xu, Giang T. Vu, Danqing Zhou, Bernard J. Koh, Roger S. McIntyre, Cyrus Ho, Roger C. Ho, Vipat Kuruchittham

**Affiliations:** 1 Institute of Cognitive Neuroscience, Faculty of Education, Huaibei Normal University, Huaibei, China; 2 College of Medicine, University of the Philippines, Manila, Philippines; 3 University Malaysia Sarawak (UNIMAS), Sarawak, Malaysia; 4 Department of Psychology, Zahedan Branch, Islamic Azad University, Zahedan, Iran; 5 College of Public Health Sciences, Chulalongkorn University, a member of Thailand One Health University Network (THOHUN), Bangkok, Thailand; 6 Institute of Clinical Psychology, University of Karachi, Karachi, Pakistan; 7 Institute for Preventive Medicine and Public Health, Hanoi Medical University, Hanoi, Vietnam; 8 Bloomberg School of Public Health, Johns Hopkins University, Baltimore, MD, United States of America; 9 DHQ Hospital Jhelum, Jhelum, Pakistan; 10 Institute for Global Health Innovations, Duy Tan University, Da Nang, Vietnam; 11 Faculty of Medicine, Duy Tan University, Da Nang, Vietnam; 12 Department of Psychology, University of Sistan and Baluchestan, Zahedan, Iran; 13 Center of Excellence in Evidence-based Medicine, Nguyen Tat Thanh University, Ho Chi Minh City, Vietnam; 14 Department of Psychological Medicine, Yong Loo Lin School of Medicine, National University of Singapore, Singapore, Singapore; 15 Mood Disorders Psychopharmacology Unit, University Health Network, University of Toronto, Toronto, Canada; 16 Department of Psychological Medicine, National University Health System, Singapore, Singapore; 17 Institute for Health Innovation and Technology (iHealthtech), Yong Loo Lin School of Medicine, National University of Singapore, Singapore, Singapore; 18 Southeast Asia One Health University Network (SEAOHUN), Chiang Mai, Thailand; Jouf University, Kingdom of Saudi Arabia, SAUDI ARABIA

## Abstract

The coronavirus disease (COVID-19) pandemic has impacted the economy, livelihood, and physical and mental well-being of people worldwide. This study aimed to compare the mental health status during the pandemic in the general population of seven middle income countries (MICs) in Asia (China, Iran, Malaysia, Pakistan, Philippines, Thailand, and Vietnam). All the countries used the Impact of Event Scale–Revised (IES-R) and Depression, Anxiety and Stress Scale (DASS-21) to measure mental health. There were 4479 Asians completed the questionnaire with demographic characteristics, physical symptoms and health service utilization, contact history, knowledge and concern, precautionary measure, and rated their mental health with the IES-R and DASS-21. Descriptive statistics, One-Way analysis of variance (ANOVA), and linear regression were used to identify protective and risk factors associated with mental health parameters. There were significant differences in IES-R and DASS-21 scores between 7 MICs (p<0.05). Thailand had all the highest scores of IES-R, DASS-21 stress, anxiety, and depression scores whereas Vietnam had all the lowest scores. The risk factors for adverse mental health during the COVID-19 pandemic include age <30 years, high education background, single and separated status, discrimination by other countries and contact with people with COVID-19 (p<0.05). The protective factors for mental health include male gender, staying with children or more than 6 people in the same household, employment, confidence in doctors, high perceived likelihood of survival, and spending less time on health information (p<0.05). This comparative study among 7 MICs enhanced the understanding of metal health in the general population during the COVID-19 pandemic.

## Introduction

Emerging psychiatric conditions and mental well-being were identified as the tenth most frequent research topic during the COVID-19 pandemic [1]. A recent systematic review found that relatively high rates of symptoms of anxiety, depression, post-traumatic stress disorder and stress were reported in the general population and health care professionals during the COVID-19 pandemic globally [2, 3]. Asia has a number of middle income countries (MICs) that face tremendous economic challenges and limited medical resources to maintain physical and mental well-being during the pandemic [4]. This extended to North America as well, with the sudden change in economic security during COVID-19 projected to increase suicide rates [5]. During the pandemic, the Asia Pacific Disaster Mental Health Network recommended to establish a mental health agenda for Asia [6]. It is therefore important to conduct research to assess psychiatric status of Asians living in MICs to develop capacity of various health systems to respond to COVID-19. Previous studies mainly focused on mental health of individual Asian countries during the pandemic without cross comparison [7–9].

With no prior comparative study found on physical and mental health of Asians living in MICs during the COVID-19 pandemic, this study aimed to investigate the impact of the pandemic on physical and mental health in 7 Asian MICs (China, Iran, Malaysia, Pakistan, Philippines, Thailand and Vietnam), identify differences among countries, understand their concerns and precautions toward COVID-19, as well as to identify protective and risk factors associated with mental health outcomes.

## Methodology

### Study design and study population

This was a cross-sectional study that involved seven countries. The recruitment was conducted after COVID-19 became an epidemic in each country. To minimize risks of COVID-19 infection, a respondent-driven sampling strategy on recruiting the general public was utilized where new participants were electronically invited by existing study respondents rather than face-to-face interaction. The respondents completed the questionnaires through an online survey platform (‘SurveyStar’, Changsha Ranxing Science and Technology in China, SurveyMonkey in Philippines, and Google Forms in other countries).

### Ethics approval

The study was approved by the Institutional Review Boards from each MIC, China (Huaibei Normal University of China, HBU-IRB-2020-001/002), Iran (Islamic Azad University, Protocol Number: IRB-2020-001), Malaysia (Universiti Malaysia Sarawak, UNIMAS/NC-21.02/03-02 Jld.4 (85)), Pakistan (University of Karachi Protocol Number: ICP-1 (101) 2698), Philippines (University of Philippines Manila Research Ethics Board, UPMREB 2020-198-01), Thailand (Chulalongkorn University, COA No. 147/2563), and Vietnam (Hanoi Medical University, QD 75/QD-YHDP&YHDP). All IRBs allowed participants aged 12 years to 17 years to participate in this study and provide their own consent because the online survey did not pose any risk to research participants. All respondents provided informed consent. Confidentiality was maintained because no personally identifiable information was collected.

### Measures and instruments

The COVID-19 online questionnaire designed by the National University of Singapore [10] had five sections: demographic, physical symptoms related to COVID-19 in the past 14 days, knowledge and concerns about COVID-19, precautionary measures against COVID, and views of health information required. Psychometric properties of the questionnaire were established in the initial phase and peak of the COVID-19 epidemic [8, 9].

The psychological impact of COVID-19 was measured using the well-validated Impact of Event Scale-Revised (IES-R) in the Asians for determining the extent of psychological impact after exposure to a traumatic event (i.e., the COVID-19 pandemic) within one week of exposure [11–14]. In this study, the Cronbach’s alpha for different versions of IES-R is very high in all countries and ranges from 0.912–0.950. Cronbach’s alpha of 0.70 or higher in measuring the internal consistency is considered “acceptable” in most social science research [15].

The mental health status of respondents was measured using the Depression, Anxiety and Stress Scale (DASS-21) [16], which has been used to assess mental health in Asians [17, 18]. Furthermore, DASS-21 assessed three domains (i.e. anxiety, depression and stress) and its psychometric properties was validated across clinical and non-clinical samples in different cultures and languages during the COVID-19 pandemic [19]. In this study, the Cronbach’s alpha (internal consistency) for different versions of DASS-21 is as follows: stress scale ranges from 0.839–0.934, anxiety scale ranges from 0.784–0.914, and depression scale ranges from 0.878–0.943. The IES-R and DASS-21 scales were previously used in research related to the COVID-19 epidemic [8, 12, 20, 21]. The DASS and IES-R questionnaires are available in the public domain, and so permission is not required to use these two questionnaires [22, 23].

### Statistical analysis

Descriptive statistics were calculated to compare demographic characteristics, physical symptoms and health service utilization, contact history, knowledge and concern, precautionary measure and additional health information variables among 7 MICs. One-Way analysis of variance (ANOVA) was calculated to compare the mean IES-R and DASS-21 scores between 7 MICs in order to determine whether the associated population mean IES-R or DASS-21 scores were significantly different. If there were significant differences among 7 MICs, the Least Significant Difference (LSD) would calculate the smallest significant between mean scores of two countries with different combinations. Any difference larger than the LSD is considered a significant result. We used linear regressions to calculate the univariate associations between independent and dependent variables including the IES-S score and DASS-21 stress, anxiety and depression subscale scores for all respondents separately. All tests were two-tailed, with a significance level of *p* <0.05. Statistical analysis was performed on IBM SPSS Statistics version 21.0.

## Results

**Fig 1:**
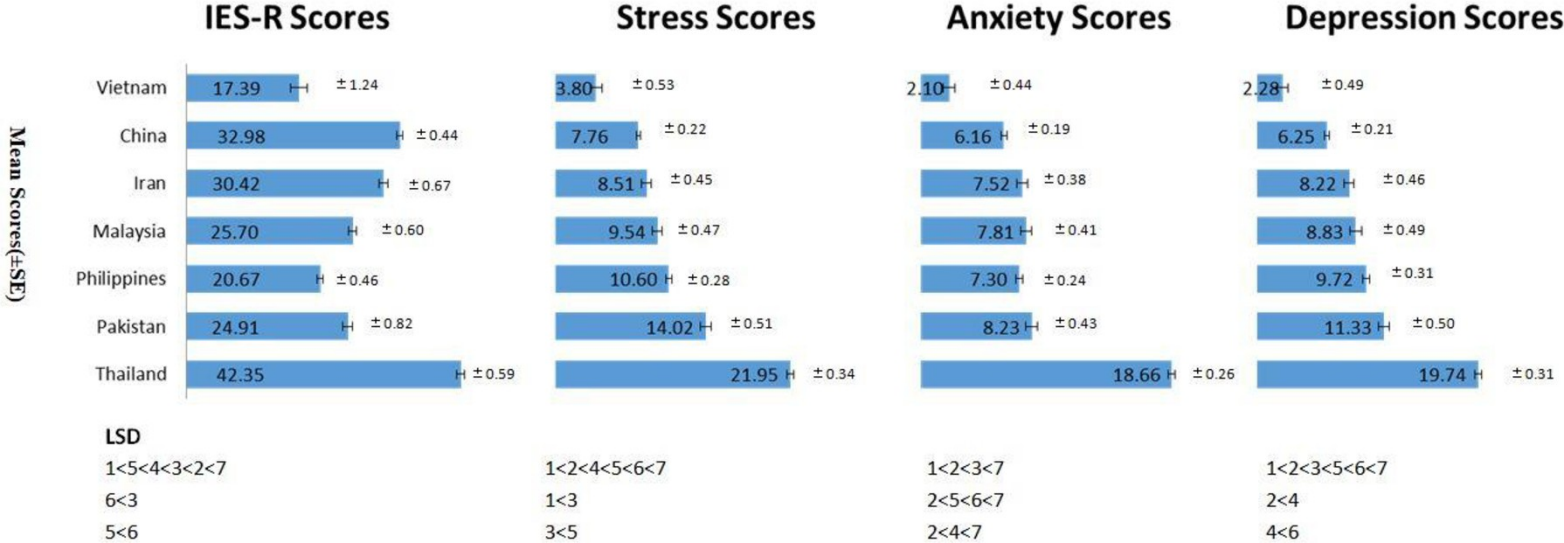
Comparison of Impact of Event Scale (Revised) IES-R and Depression, Anxiety and Stress Scale– 21 (DASS-21) scores among 7 middle-income countries in Asia (1: Vietnam, 2: China, 3: Iran, 4: Malaysia, 5: Philippines, 6: Pakistan, 7: Thailand).

A total of 4479 participants from 7 MICs in Asia completed the survey. The distribution of the number of participants by country is listed as follows: China (27%), Philippines (19%), Malaysia (16.2%), Iran (12.3%), Thailand (11.6%), Pakistan (11.3%), and Vietnam (2.7%). Fig 1 compares the IES-R and DASS-21 scores amongst all 7 MICs in Asia.

The top three countries with highest IES-R scores were Thailand (mean 42.35, SD 13.39), China (mean 32.98, SD 15.42), and Iran (mean 30.42, SD 15.82). The top three countries with highest DASS-21 stress scores were Thailand (mean 21.94, SD 7.74), Pakistan (mean 14.02, SD 11.53) and Philippines (mean 10.60, SD 8.01). The top three countries with highest DASS-21 anxiety scores were Thailand (mean 18.66, SD 5.98), Pakistan (mean 8.23, SD 9.69) and Malaysia (mean 7.80, SD 10.95). The top three countries with highest DASS-21 depression scores were Thailand (mean 19.74, SD 6.99), Pakistan (mean 11.33, SD 11.28) and Philippines (mean 9.72, SD 8.99).

Differences in IES-R scores and DASS-21 stress, anxiety, depression scores amongst the 7 MICs were all statistically significant (IES-R: F(6, 4472) = 144.47, p<0.001, η2 = 0.16; Stress: F(6,4472) = 167.49 p<0.001, η2 = 0.18; Anxiety: F (6,4471) = 172.03, p<0.001, η2 = 0.19; Depression: F(6, 4472) = 137.11, p<0.001, η2 = 0.16). Vietnam had the lowest scores of IES-R (mean 17.39, SD 13.72), stress (mean 3.80, SD 5.81), anxiety (mean 2.10, SD 4.91) and depression (mean 2.28, SD 5.43). The LSD analysis revealed that the scores of Vietnam were significantly lower than the other countries (p<0.05).

S1 Table compares the demographics of 7 MICs. More than half of participants were women in all countries (Range: 52.6% in Pakistan to 76.8% in Thailand). More than half of Chinese, Filipino, Iranian and Pakistani participants were below age of 31 years. Majority of Chinese, Vietnamese and Malaysian respondents were married while majority of Filipino and Thai respondents were single. Majority of Filipino, Iranian, Pakistani, Malaysian and Thai respondents did not have children. More than half of participants stayed in a household with more than 3–5 people across all countries except Pakistan (49%). Majority of respondents from Philippines, Pakistan, Vietnam and Malaysia were employed when the study was conducted.

Table 1 shows the association between demographic characteristics of all participants and mental health parameters. Demographic characteristics associated with lower psychological impact were male gender whereas age younger than 30 years and students were associated with higher psychological impact. Participants who have children were associated with lower stress, anxiety and depression whereas participants with higher education, single and separated status were associated with higher stress, anxiety and depression. Staying with 6 or more people and those who were employed were associated with lower anxiety and depression.

**Table 1:** Comparison of the association between demographic characteristics of participants in seven Asian countries and psychological impact as well as adverse mental health status (N = 4479).

Variable	Impact of event			Stress			Anxiety			Depression		
	*R*^*2*^	*AR*^*2*^	*B (95%CI)*	*R*^*2*^	*AR*^*2*^	*B (95%CI)*	*R*^*2*^	*AR*^*2*^	*B (95%CI)*	*R*^*2*^	*AR*^*2*^	*B (95%CI)*
**Gender**												
Male	0.006	0.006	-0.22***(-0.30 to -0.13)	<0.001	<0.001	-0.04 (-0.10 to 0.03)	<0.001	<0.001	-0.04(-0.13 to 0.05)	<0.001	<0.001	-0.04(-0.12 to 0.04)
Female			Reference			Reference			Reference			Reference
**Age**												
12–21 years	0.014	0.013	0.28***(0.15 to 0.42)	0.003	0.003	0.01(-0.10 to 0.12)	0.002	0.002	-0.12(-0.27 to 0.03)	0.007	0.006	0.13(-0.004 to 0.27)
22–30 years			0.35***(0.23 to 0.48)			0.09(-0.02 to 0.19)			-0.03(-0.17 to 0.11)			0.11(-0.02 to 0.24)
31–40 years			0.05(-0.09 to 0.20)			0.01(-0.11 to 0.13)			-0.15(-0.31 to 0.01)			-0.10(-0.24 to 0.05)
41–49 years			-0.01(-0.16 to 0.14)			-0.11(-0.24 to 0.02)			-0.22*(-0.39 to -0.05)			-0.14(-0.30 to 0.01)
50 years and above			Reference			Reference			Reference			Reference
**Education Level**												
Degree holder	0.001	<0.001	-0.03(-0.21 to 0.15)-	0.003	0.003	0.28***(0.13 to 0.43))	0.002	0.001	0.27**(0.07 to 0.48)	0.003	0.002	0.31**(0.12 to 0.49)
High School			0.15(-0.37 to 0.06)			0.27**(0.09 to 0.45			0.26*(0.02 to 0.50)			0.36**(0.15 to 0.58)
Secondary or below			Reference			Reference			Reference			Reference
**Marital Status**												
Single	0.001	<0.001	-0.06(-0.14 to 0.01)	0.018	0.018	0.29***(0.23 to 0.35)	0.015	0.014	0.34***(0.25 to 0.42)	0.034	0.033	0.49***(0.41 to 0.56)
Married			-0.03(-0.34 to 0.27)			0.34**(0.08 to 0.59)			0.57**(0.23 to 0.91)			0.46**(0.16 to 0.77)
Divorced or Separated			0.15(-0.26 to 0.56)			0.33(-0.01 to 0.66)			0.51*(0.06 to 0.97)			0.40(-0.01 to 0.80)
Widowhood			Reference			Reference			Reference			Reference
**Parental Status**												
Has children	<0.001	<0.001	0.001(-0.08 to 0.08)	0.032	0.031	-0.38***(-0.45 to -0.32)	0.027	0.026	-0.48***(-0.56 to -0.39)	0.046	0.046	-0.57***(-0.64 to -0.49)
No children			Reference			Reference			Reference			Reference
**Family Size**												
6 people and above	0.006	0.005	-0.11(-0.31 to 0.08)	<0.001	-0.001	-0.02(-0.18 to 0.14)	0.003	0.002	-0.30**(-0.52 to -0.09)	0.002	0.002	-0.27**(-0.47 to -0.07)
3–5 people			0.12(-0.06 to 0.31)			-0.002(-0.16 to 0.15)			-0.16(-0.37 to 0.05)			-0.15(-0.34 to 0.04)
2 people			0.03(-0.20 to 0.26)			-0.03(-0.22 to 0.16)			-0.13(-0.38 to 0.13)			-0.18(-0.41 to 0.06)
1 person			Reference			Reference			Reference			Reference
**Employment Status**												
Unemployed	0.020	0.019	0.11(-0.16 to 0.38)	0.008	0.007	0.01(-0.22 to 0.24)	0.011	0.010	-0.22(-0.52 to 0.09)	0.023	0.022	-0.05(-0.32 to 0.23)
Housewife			-0.05(-0.33 to 0.22)			-0.05(-0.28 to 0.18)			-0.24(-0.55 to 0.07)			-0.21(-0.49 to 0.07)
Student			0.27*(0.04 to 0.49)			0.06(-0.13 to 0.25)			-0.08(-0.34 to 0.17)			0.07(-0.16 to 0.30)
Employed			-0.12(-0.34 to 0.11)			-0.14(-0.33 to 0.04)			-0.40**(-0.65 to -0.15)			-0.35**(-0.58 to -0.12)
Retired			Reference			Reference			Reference			Reference

* *p*<0.05;

** *p*<0.01,

*** *p*<0.001

S2 Table shows the frequency of physical symptoms that resemble COVID-19 infection and there were significant differences among all countries. During the COVID-19 pandemic, the most common physical symptoms reported by general population in the 7 countries were headache (23.13%), cough (21.86%) and sore throat (19.29%). About 8.13% of respondents consulted General Practitioner (GP); 2.69% were hospitalized; 3.89% were tested positive for COVID-19 and 57.1% had a health insurance. Pakistani had the significantly highest proportion of respondents consulted GP (27.5%), hospitalized (16.4%), receiving COVID-19 test (17.2%) and being isolated (17.8%). Table 2 shows the association between physical symptoms and mental health outcomes. The physical symptoms that were significantly associated with higher scores in all mental health outcomes (IES-R and DASS-21 subscales) including rhinitis and persistent fever with cough or breathing difficulties. Chills or rigors, headache and nausea or vomiting were associated with higher DASS-21 stress and anxiety scores. Myalgia, cough, dizziness and sore throat were associated with higher score of IES-R. Usage of medical services such as seeing a doctor, hospitalization, recent COVID-19 testing, quarantine, poor rating of health status that were significantly associated with higher scores in all mental health outcomes (IES-R and DASS-21 subscales). History of chronic illness were significantly associated with higher DASS-21 subscale scores. Having medical insurance coverage was associated with higher IES-R scores.

**Table 2:** Association between physical health status in the past 14 days and the psychological impact of the 2019 coronavirus disease (COVID-19) outbreak as well as adverse mental health status during the epidemic (N = 4479).

Variable	Impact of Event			Stress			Anxiety			Depression		
	*R*^*2*^	*AR*^*2*^	*B (95%CI)*	*R*^*2*^	*AR*^*2*^	*B (95%CI)*	*R*^*2*^	*AR*^*2*^	*B (95%CI)*	*R*^*2*^	*AR*^*2*^	*B (95%CI)*
**Prolonged or Recuring Fever**												
Yes	0.001	0.001	0.10(-0.01 to 0.21)	<0.001	<0.001	0.004(-0.09 to 0.09)	<0.001	<0.001	0.03(-0.09 to 0.16)	<0.001	<0.001	0.08(-0.03 to 0.19)
No			Reference			Reference			Reference			Reference
**Chills or Rigors**												
Yes	0.001	<0.001	0.09(-0.02 to 0.20)	0.001	0.001	-0.11*(-0.20 to- 0.02)	0.001	0.001	-0.14*(-0.26 to -0.01)	<0.001	<0.001	-0.08(-0.19 to 0.04)
No			Reference			Reference			Reference			Reference
**Headache**												
Yes	<0.001	<0.001	0.02(-0.07 to 0.11)	0.002	0.002	0.11**(0.04 to 0.19)	0.002	0.002	0.15**(0.05 to 0.25)	0.002	0.001	0.13**(0.03to 0.22)
No			Reference			Reference			Reference			Reference
**Myalgia**												
Yes	0.003	0.003	0.18***(0.08 to 0.28)	<0.001	<0.001	0.03(-0.06 to 0.11)	<0.001	<0.001	0.08(-0.03 to 0.19)	<0.001	<0.001	0.03(-0.07 to 0.13)
No			Reference			Reference			Reference			Reference
**Cough**												
Yes	0.004	0.003	0.19***(0.10 to 0.28)	<0.001	<0.001	0.04(-0.04 to 0.12)	0.001	0.001	0.12*(0.02 to 0.23)	<0.001	<0.001	0.07(-0.03 to 0.16)
No			Reference			Reference			Reference			Reference
**Breathing Difficulties**												
Yes	0.001	<0.001	0.10(-0.01 to 0.21)	<0.001	<0.001	0.02(-0.07 to 0.11)	<0.001	<0.001	0.05(-0.07 to 0.18)	<0.001	<0.001	0.08(-0.04 to 0.19)
No			Reference			Reference			Reference			Reference
**Dizziness**												
Yes	0.003	0.003	0.20***(0.10 to 0.30)	<0.001	<0.001	-0.01(-0.09 to 0.08)	<0.001	<0.001	0.02(-0.10 to 0.13)	<0.001	<0.001	0.01(-0.10 to 0.11)
No			Reference			Reference			Reference			Reference
**Rhinitis**												
Yes	0.002	0.002	0.14**(0.04 to 0.24)	0.003	0.003	-0.16***(-0.24 to- 0.08)	0.001	0.001	-0.14*(-0.25 to -0.03)	0.001	0.001	-0.12*(-0.22 to- 0.02)
No			Reference			Reference			Reference			Reference
**Sore Throat**												
Yes	0.002	0.002	0.15**(0.05to 0.24)	<0.001	<0.001	0.06(-0.02 to 0.14)	0.001	0.001	0.14*(0.03 to 0.25)	<0.001	<0.001	0.07(-0.03 to 0.17)
No			Reference			Reference			Reference			Reference
**Recurrent fever with cough or breathing difficulties**												
Yes	0.003	0.003	0.65***(0.31 to 1.00)	0.016	0.015	1.22***(0.93 to 1.50)	0.020	0.019	1.84***(1.46 to 2.22)	0.018	0.017	1.58***(1.23 to 1.93)
No			Reference			Reference			Reference			Reference
**Nausea, vomiting and diarrhoea**												
Yes	<0.001	<0.001	0.01(-0.10 to 0.13)	0.002	0.001	-0.13**(-0.23 to -0.04)	0.002	0.002	-0.19**(-0.31 to -0.06)	0.001	<0.001	-0.10(-0.22 to 0.02)
No			Reference			Reference			Reference			Reference
**Have you seen a doctor or visited a GP?**												
Yes	0.003	0.003	0.25***(0.11 to 0.39)	0.023	0.023	0.59***(0.48 to 0.71)	0.022	0.022	0.79***(0.63 to 0.94)	0.022	0.022	0.71***(0.57 to 0.85)
No			Reference			Reference			Reference			Reference
**Hospitalisation**												
Yes	0.006	0.006	0.61***(0.38 to 0.84)	0.034	0.034	1.23***(1.04 to 1.42)	0.041	0.040	1.81***(1.55 to 2.06)	0.038	0.038	1.59***(1.36 to1.82)
No			Reference			Reference			Reference			Reference
**Have you been tested for COVID-19**												
Yes	0.005	0.004	0.45***(0.26 to 0.65)	0.024	0.024	0.87***(0.70 to 1.03)	0.025	0.025	1.19***(0.98 to 1.41)	0.027	0.026	1.11***(0.91 to 1.30)
No			Reference			Reference			Reference			Reference
**Have you been quarantined**												
Yes	0.004	0.004	0.41***(0.23 to 0.60)	0.015	0.015	0.65***(0.50 to 0.80)	0.014	0.013	0.83***(0.63 to 1.04)	0.016	0.016	0.82***(0.63 to 1.01)
No			Reference			Reference			Reference			Reference
**Self-assessment of health status**												
Very bad or bad	0.026	0.026	0.48*(0.12 to 0.84)	0.093	0.092	1.23***(0.94 to 1.51)	0.113	0.113	1.41***(1.02 to 1.80)	0.108	0.108	1.40***(1.05 to 1.75)
Normal			0.55***(0.45 to 0.65)			0.82***(0.74 to 0.90)			1.27***(1.16 to 1.37)			1.11***(1.01to 1.21)
Good or very good			Reference			Reference			Reference			Reference
**History of chronic disease**												
Yes	<0.001	<0.001	0.07(-0.03 to 0.18)	0.011	0.010	0.31***(0.22 to 0.39)	0.015	0.014	0.49***(0.37 to 0.60)	0.008	0.008	0.33***(0.22 to 0.43)
No			Reference			Reference			Reference			Reference
**Health insurance**												
Yes	0.017	0.016	0.34***(0.26 to 0.41)	<0.001	<0.001	-0.02(-0.08 to 0.05)	0.001	0.001	0.08(-0.004 to 0.17)	<0.001	<0.001	-0.02(-0.10 to 0.05)
No			Reference			Reference			Reference			Reference

* *p*<0.05;

** *p*<0.01,

*** *p*<0.001

S3 Table shows the belief of route of transmission among participants in 7 MICs and there were significant differences among all countries. Out of all participants, there were a small number of participants who did not agree with transmission of COVID-19 being via droplets (10.34%) and contaminated objects (17.21%). It is interesting to note that China (60.5%) and Vietnam (59.8%) demonstrated significantly higher percentage of participants who believed in airborne transmission compared to .64.76% of participants from the other five countries who did not agree that COVID-19 was airborne transmitted.

Participants expressing confident and very confident in their doctors diagnosing COVID-19 were very high in Malaysia (93.8%) and China (92.9%); level of confidence was much lower in Iran (65.5%) and Pakistan (62.6%). About 50.26% of participants reported that they were likely and very likely to contract COVID-19, with Malaysian participants demonstrating the highest perceived risk of COVID-19 (72.8%) whilst the Filipino demonstrated the highest proportion of participants believing that they would not contract COVID-19 (53.2%). About 89.8% of Thai participants believed that they would survive if contracted with COVID-19 while the Pakistani had the highest proportion who believed that they would not survive COVID-19 (15.4%). About 78.43% of participants were satisfied with health information related to COVID-19; Vietnamese participants reported the highest proportion of satisfaction (97.5%). About 77.38% of participants were worried their family members contracting COVID-19. Pakistani participants reported the highest proportion of people who faced discrimination (42.7%). About 44.68% of participants spent more than 2 hours per day to view information on COVID-19 with Filipino participants having the highest proportion for spending more than 2 hours per day to view information (47.2%).

Table 3 shows the association between knowledge and concerns related to COVID-19 and mental health parameters. Agreement with airborne, contact with contaminated objects and droplet transmission was associated with higher DASS-21 in all subscales. Likelihood of contracting COVID-19, discrimination against by other countries and contact with people infected with COVID-19 were associated with higher IES-R or DASS-21 scores. Confidence in one’s own doctor diagnosing COVID-19, high likelihood of survival if infected with COVID-19 and spent less than two hours per day to monitor information relating to COVID-19 were associated with lower level of IES-R or DASS-21 scores.

**Table 3:** Association between Knowledge and belief about COVID-19 and the psychological impact of the 2019 coronavirus disease (COVID-19) outbreak as well as adverse mental health status during the epidemic (N = 4479).

Variable	Impact of Event			Stress			Anxiety			Depression		
	*R*^*2*^	*AR*^*2*^	*B (95%CI)*	*R*^*2*^	*AR*^*2*^	*B (95%CI)*	*R*^*2*^	*AR*^*2*^	*B (95%CI)*	*R*^*2*^	*AR*^*2*^	*B (95%CI)*
**Mode of transmission of COVID-19**												
***Droplets***												
Agree	<0.001	<0.001	-0.09(-0.21 to 0.04)	0.003	0.003	0.19***(0.09 to 0.29)	0.002	0.002	0.24**(0.10 to 0.38)	0.003	0.003	0.23***(0.10 to 0.35)
Disagree or Uncertain			Reference			Reference			Reference			Reference
***Contact with contaminated surfaces***												
Agree	0.001	<0.001	-0.08(-0.18 to 0.02)	0.002	0.001	0.11**(0.03 to 0.20)	0.001	0.001	0.13*(0.02 to 0.25)	0.003	0.003	0.20***(0.09 to 0.30)
Disagree or Uncertain			Reference			Reference			Reference			Reference
***Airborne***												
Agree	<0.001	<0.001	-0.003(-0.08 to 0.07)	0.010	0.009	-0.21***(-0.28 to -0.15)	0.010	0.010	-0.30***(-0.38 to -0.21)	0.014	0.014	-0.32***(-0.39 to -0.24)
Disagree or Uncertain			Reference			Reference			Reference			Reference
**Level of confidence in one’s own doctor to diagnose or recognize COVID-19**												
Very confident	0.012	0.011	-0.08(-0.22 to 0.07)	0.038	0.037	-0.19**(-0.31 to -0.07)	0.040	0.039	-0.21*(-0.37 to -0.05)	0.045	0.045	-0.34***(-0.49 to -0.20)
Somewhat confident			0.16*(0.02 to 0.31)			0.04(-0.07 to 0.16)			0.19*(0.03 to 0.35)			-0.03(-0.17 to 0.11)
Not very confident			0.35***(0.17 to 0.52)			0.54***(0.40 to 0.69)			0.77***(0.58 to 0.97)			0.63***(0.46 to 0.81)
Not confident or uncertain			Reference			Reference			Reference			Reference
**Likelihood of contracting COVID-19 during the pandemic**												
Very possible	0.011	0.010	0.36***(0.24 to 0.48)	0.036	0.036	0.47***(0.37 to 0.57)	0.052	0.052	0.74***(0.61 to 0.87)	0.039	0.038	0.56***(0.44 to 0.68)
Somewhat possible			0.15**(0.04 to 0.26)			0.24***(0.15 to 0.33)			0.37***(0.25 to 0.48)			0.30***(0.19 to 0.40)
Not very possible			0.08(-0.03 to 0.19)			0.02(-0.07 to 0.11)			0.08(-0.04 to 0.21)			0.13*(0.01 to 0.24)
Impossible			0.20**(0.07 to 0.32)			0.30***(0.20 to 0.40)			0.58***(0.44 to 0.71)			0.51***(0.39 to 0.63)
Uncertain			Reference			Reference			Reference			Reference
**Likelihood of survival after contracting COVID-19**												
Very possible	0.016	0.015	-0.28**(-0.54 to -0.03)	0.009	0.008	-0.44***(0.65 to -0.22)	0.006	0.006	-0.280.(-0.58 to 0.01)	0.009	0.009	-0.52***(-0.78 to -0.26)
Somewhat possible			0.04 (-0.22 to 0.29)			-0.34***(-0.55 to -0.12)			-01.2(-0.41 to 0.17)			-0.40**(-0.65 to -0.14)
Not very possible			0.19(-0.09 to 0.47)			-0.11(-0.36 to 0.13)			0.14(-0.19 to 0.46)			-0.11 (-0.40 to 0.18)
Impossible			Reference			Reference			Reference			Reference
**Level of satisfaction with the amount of health information available regarding COVID-19**												
Very satisfied	0.018	0.017	-0.44***(-0.59 to -0.28)	0.017	0.016	-0.40***(-0.53 to -0.27)	0.021	0.020	-0.59***(-0.77 to -0.42)	0.027	0.027	-0.59***(-0.75 to -0.43)
Somewhat satisfied			-0.31***(-0.46 to -0.16)			-0.22***(-0.35 to -0.10)			-0.28**(-0.45 to -0.11)			-0.28***(-0.43 to -0.12)
Not very satisfied			0.06(-0.11 to 0.23)			-0.02(-0.16 to 0.13)			-0.001(-0.19 to 0.19)			0.04(-0.13 to 0.22)
Unsatisfied or uncertain			Reference			Reference			Reference			Reference
**Level of worry about family members being diagnosed with COVID-10**												
Very worried	0.017	0.016	0.13(-0.15 to 0.42)	0.044	0.043	-0.11(-0.35 to 0.13)	0.051	0.050	-0.27(-0.59 to 0.05)					0.045	0.044	-0.29*(-0.58 to -0.003)
Somewhat worried			0.01(-0.28 to 0.30)			-0.37**(-0.61 to -0.13)			-0.58***(-0.90 to -0.26)	-0.54***(-0.83 to -0.25)					
Not very worried			0.33*(0.03 to 0.63)			0.10(-0.15 to 0.34)			0.14(-0.20 to 0.47)	0.09(-0.22 to 0.39)					
Not worried			0.63***(0.31 to 0.95)			0.46**(0.20 to 0.72)			0.60**(0.25 to 0.95)	0.45**(0.13 to 0.76)					
No family members			Reference			Reference			Reference	Reference					
**Did you feel discriminated against by other countries after the outbreak?**												
Yes	0.005	0.004	0.25***(0.13 to 0.36)	0.067	0.067	0.76***(0.67 to 0.85)	0.041	0.041	0.82***(0.69 to 0.95)	0.054	0.054	0.84***(0.73 to 0.95)
No			Reference			Reference			Reference			Reference
**Time spent on monitoring information regarding COVID-19**												
Less than 1 hour	0.061	0.060	-0.83***(-0.96 to -0.69)	0.002	0.002	-0.03 (-0.13 to 0.07)	0.004	0.003	-0.24**(-0.38 to -0.10)	0.004	0.003	-0.03(-0.16 to 0.10)
1–2 hours			-0.63***(-0.75 to -0.50)			0.13**(0.04 to 0.23)			-0.12(-0.25 to 0.02)			0.21**(0.08 to 0.33)
2 hours and above			Reference			Reference			Reference			Reference
**Direct contact with people infected by COVID-19**												
Yes	<0.001	<0.001	0.14(-0.10 to 0.39)	0.012	0.012	0.75***(0.55 to 0.95)	0.010	0.010	0.94***(0.67 to 1.21)	0.010	0.010	0.87***(0.62 to 1.11)
No			Reference			Reference			Reference			Reference
**Indirect contact with people infected by COVID-19**												
Yes	<0.001	<0.001	-0.06(-0.27 to 0.15)	0.008	0.008	0.53***(0.36 to 0.71)	0.008	0.008	0.73***(0.50 to 0.96)	0.006	0.006	0.57*** (0.36 to 0.78)
No			Reference			Reference			Reference			Reference
**Contact with materials contaminated by COVID-19**												
Yes	0.003	0.003	0.41***(0.18 to 0.63)	0.021	0.021	0.94***(0.75 to 1.12)	0.022	0.022	1.28***(1.03 to 1.53)	0.022	0.022	1.16***(0.93 to 1.39)
No			Reference			Reference			Reference			Reference

* *p*<0.05;

**p<0.01;

***p<0.001

S4 Table shows the prevalence of precautionary measures and there were significant differences among 7 MICs (p<0.001). High percentages were reported by participants covering their mouth and nose after sneezing (98.0%), avoided sharing utensils (90.8%), practised hand hygiene (98.9%), washed hand after touching contaminated objects (96.2%), and wear face masks (93.5%). All Vietnamese participants (100%) responded wearing a face mask. About 68% of respondents felt that people were too worried about COVID-19 with Malaysia (90.5%), Thailand (90.5%) and Pakistan (86.6%) as the top three countries. Approximately 53% of respondents spent 20–24 hours per day at home; with China (84.7%), Iran (73.5%) and Philippines (55%) as the top three countries.

Table 4 shows the association between precautionary measures related to COVID-19 and mental health parameters. Avoidance of sharing cutlery dealing meals was associated with higher anxiety and depression. In contrast, hand hygiene practice was associated with lower IES-R and DASS-21 in all subscales. Wearing a face mask was associated with lower levels of stress and depression. Worries about COVID-19 was associated with significantly higher levels of DASS-21 in all subscales. Shorter duration of homestay was associated with higher levels of anxiety, depression and stress as compared to those who stayed at home for 20–24 hours per day.

**Table 4:** Association between precautionary measures and the psychological impact of the 2019 coronavirus disease (COVID-19) outbreak as well as adverse mental health status during the epidemic (N = 4479).

Variable	Impact of event			Stress			Anxiety			Depression		
	*R*^*2*^	*AR*^*2*^	*B (95%CI)*	*R*^*2*^	*AR*^*2*^	*B (95%CI)*	*R*^*2*^	*AR*^*2*^	*B (95%CI)*	*R*^*2*^	*AR*^*2*^	*B (95%CI)*
**Covering mouth after coughing or sneezing**												
Yes	<0.001	<0.001	-0.11(-0.38 to 0.16)	0.001	<0.001	0.19(-0.04 to 0.41)	<0.001	<0.001	0.14(-0.17 to 0.44)	0.001	<0.001	0.21(-0.06 to 0.49)
No			Reference			Reference			Reference			Reference
**Avoidance of sharing cutlery during meals**												
Yes	<0.001	<0.001	0.08(-0.06 to 0.21)	0.001	<0.001	0.09(-0.02 to 0.20)	0.001	0.001	0.17*(0.02 to 0.31)	0.001	0.001	0.15*(0.02 to 0.29)
No			Reference			Reference			Reference			Reference
**Washing your hands using soap or hand sanitizer**												
Yes	0.002	0.001	-0.49**(-0.85 to -0.13)	<0.001	<0.001	-0.02(-0.32 to 0.28)	<0.001	<0.001	-0.22(-0.62 to 0.19)	<0.001	<0.001	-0.12(-0.48 to 0.25)
No			Reference			Reference			Reference			Reference
**Washing hands immediately after coughing, sneezing or rubbing your nose**												
Yes	0.001	0.001	-0.22*(-0.42 to -0.03)	0.002	0.002	-0.23**(-0.40 to -0.07)	0.004	0.003	-0.46***(-0.68 to -0.23)	0.002	0.001	-0.27**(-0.48 to -0.07)
No			Reference			Reference			Reference			Reference
**Wearing a face mask**												
Yes	0.001	0.001	0.15(-0.002 to 0.30)	0.002	0.001	-0.17**(-0.30 to -0.05)	<0.001	<0.001	-0.01(-0.18 to 0.17)	0.003	0.002	-0.28***(-0.43 to -0.12)
No			Reference			Reference			Reference			Reference
**Washing hands after coming into contact with contaminated surfaces**												
Yes	0.001	<0.001	-0.27(-0.60 to 0.06)	0.001	0.001	-0.31*(-0.58 to -0.04)	0.003	0.002	-0.64**(-1.00 to -0.27)	0.003	0.002	-0.58**(-0.91 to -0.25)
No			Reference			Reference			Reference			Reference
**Are people too worried about COVID-19?**												
Yes	<0.001	<0.001	-0.03(-0.11 to 0.05)	0.012	0.012	0.25***(0.18 to 0.32)	0.016	0.016	0.39***(0.30 to 0.48)	0.014	0.014	0.33***(0.25 to 0.41)
No			Reference			Reference			Reference			Reference
**Time spent at home**												
0–10 hours	0.010	0.009	-0.29***(-0.40 to -0.17)	0.031	0.030	0.23***(0.14 to 0.32)	0.026	0.025	0.23***(0.11 to 0.36)	0.023	0.023	0.19**(0.08 to 0.30)
10–20 hours			0.12*(0.01 to 0.22)			0.46***(0.37 to 0.54)			0.59***(0.47 to 0.71)			0.51***(0.40 to 0.61)
20–24 hours			Reference			Reference			Reference			Reference

* *p*<0.05;

**p<0.01;

***p<0.001

S5 Table compares the health information needs of participants from 7 MICs and there were significant differences among 7 MICs. The Chinese had the highest proportion who wanted to understand the symptoms of COVID-19 (91.6%), the prevention method (93.7%), effectiveness of drugs and vaccines (94.1%), number of infected cases and location (95.9%), travel advice (96.9%), mode of transmission (94.5%), required regular information update (92.7%) and personalized information (96.8%). The Iranians had the highest proportion who sought advices regarding treatment methods (90.4%) and Malaysians had the highest proportion who wanted to understand local outbreaks (94.2%).

Table 5 shows the association between health information needs about COVID-19 and mental health parameters. Most additional information including information on COVID-19 symptoms, prevention, treatment advice, needs for regular updates, knowledge on local transmission, effectiveness on drugs and vaccines, number of infected people based on geographical locations, travel advice and transmission mode of COVID were associated with higher IES-R scores. In contrast, the need for more personalized information, information on the effectiveness of drugs and vaccines, travel advices, transmission mode were associated with significantly lower level of depression.

**Table 5:** Comparison of the associations between information needs about COVID-19, psychological impact and mental health status in participants of the seven MICs (N = 4479).

Variable	Impact of event			Stress			Anxiety			Depression		
	*R*^*2*^	*AR*^*2*^	*B (95%CI)*	*R*^*2*^	*AR*^*2*^	*B (95%CI)*	*R*^*2*^	*AR*^*2*^	*B (95%CI)*	*R*^*2*^	*AR*^*2*^	*B (95%CI)*
**Understanding of symptoms related to COVID-19**												
Yes	0.025	0.025	0.49***(0.40 to 0.58)	<0.001	<0.001	-0.02(-0.09 to 0.06)	0.001	<0.001	0.09(-0.01 to 0.19)	<0.001	<0.001	-0.06(-0.16 to 0.03)
No			Reference			Reference			Reference			Reference
**Prevention advice**												
Yes	0.026	0.026	0.51***(0.42 to 0.60)	<0.001	<0.001	0.04(-0.04 to 0.11)	0.001	0.001	0.11*(0.01 to 0.21)	<0.001	<0.001	-0.04(-0.14 to 0.05)
No			Reference			Reference			Reference			Reference
**Treatment advice**												
Yes	0.015	0.014	0.38***(0.29 to 0.47)	<0.001	<0.001	0.05(-0.03 to 0.13)	0.001	0.001	0.10(-0.002 to 0.20)	<0.001	<0.001	0.01(-0.09 to 0.10)
No			Reference			Reference			Reference			Reference
**Need for regular information updates**												
Yes	0.016	0.016	0.42***(0.33 to 0.52)	<0.001	<0.001	-0.05(-0.14 to 0.03)	<0.001	<0.001	0.02(-0.09 to 0.13)	<0.001	<0.001	-0.08(-0.18 to 0.02)
No			Reference			Reference			Reference			Reference
**Need for knowledge on local transmissions**												
Yes	0.020	0.020	0.46***(0.36 to 0.57)	0.001	0.001	-0.08(-0.16 to 0.001)	<0.001	<0.001	0.04(-0.06 to 0.15)	0.001	0.001	-0.10(-0.20 to 0.004)
No			Reference			Reference			Reference			Reference
**Need for more personalized information, such as advice for those with pre-existing medical conditions**												
Yes	0.021	0.021	0.47***(0.37 to 0.56)	<0.001	<0.001	-0.04(-0.12 to 0.03)	<0.001	<0.001	0.04(-0.07 to 0.14)	0.002	0.001	-0.13**(-0.22 to -0.03)
No			Reference			Reference			Reference			Reference
**Need to know the effectiveness of drugs and vaccines available**												
Yes	0.011	0.011	0.38***(0.27 to 0.48)	0.001	0.001	-0.11*(-0.20 to -0.03)	0.001	0.001	-0.11(-0.23 to 0.002)	0.003	0.003	-0.19***(-0.29 to -0.08)
No			Reference			Reference			Reference			Reference
**Need to know the number of people infected, and geographical location**												
Yes	0.019	0.018	0.45***(0.35 to 0.54)	<0.001	<0.001	-0.06(-0.14 to 0.02)	<0.001	<0.001	0.05 (-0.06 to 0.15)	0.001	0.001	-0.11*(-0.21 to -0.01)
No			Reference			Reference			Reference			Reference
**Need for travel advice**												
Yes	0.025	0.025	0.48***(0.39 to 0.57)	<0.001	<0.001	-0.04(-0.11 to 0.03)	<0.001	<0.001	0.07(-0.03 to 0.17)	0.002	0.001	-0.12**(-0.21 to -0.03)
No			Reference			Reference			Reference			Reference
**Need to understand transmission modes of COVID-19**											
Yes	0.024	0.024	0.50***(0.41 to 0.59)	<0.001	<0.001	-0.04(-0.12 to 0.04)	<0.001	<0.001	0.06(-0.05 to 0.16)	0.001	0.001	-0.12*(-0.22 to -0.02)
No			Reference			Reference			Reference			Reference
**Need to know other countries’ response to COVID-19**												
Yes	0.001	0.001	0.08(-0.002 to 0.16)	0.009	0.009	0.22***(0.15 to 0.28)	0.013	0.013	0.36***(0.27 to 0.45)	0.009	0.009	0.26***(0.18 to 0.35)
No			Reference			Reference			Reference			Reference

*p<0.05;

**p<0.01;

***p<0.001

## Discussion

The main findings of this first multinational population-based study in MICs in Asia during the COVID-19 pandemic are summarized as follows. First, Thai respondents reported the highest levels of IES-R and DASS-21 scores. Second, Pakistani respondents reported the second highest levels of DASS-21 scores. Comparatively, Vietnamese respondents reported the lowest levels in DASS-21 scores. Third, Iranian respondents demonstrated the lowest confidence in their doctors whilst Pakistani respondents had the highest proportion who believed they would not survive COVID-19 and reported discrimination.

Assessing COVID-19’s association with respondents’ mental health, the three most common physical symptoms associated with adverse mental health were headache, cough and sore throat. Risk factors associated with adverse mental health during the COVID-19 pandemic include age <30 years old, high education background, single and separated status, discrimination by other countries, contact with people with COVID-19 and worries about COVID-19. Protective factors for mental health during the COVID-19 pandemic include male gender, staying with children, staying with 6 or more people, employment, confidence in own’s doctors diagnosing COVID-19, high perceived likelihood of surviving COVID-19, spending less time on health information, hand hygiene practice and wearing a face mask. Importantly, these findings will be significantly helpful for healthcare administrators in Asia at the national and local community levels [24] when preparing for the next wave of COVID-19 outbreak and future pandemics [25].

Iran had the highest total reported COVID cases (386,658) and number of COVID cases per 1 million people (4,593), as well as the highest number of deaths from COVID (22,293) and deaths per 1 million people (265) [26]. Pakistan had the second highest number of cases (298,509) and deaths (6,342) [26]. Of the 7 MICs, Vietnam had the lowest total numbers and rates across all seven countries, with 1,049 reported cases, 35 deaths and rates of just 11 cases and 0.4 deaths per 1 million [26]. As a result, Vietnamese respondents reported the lowest IES-R and DASS-21 scores. Vietnam has adopted several strategies to combat COVID-19 including development of the action plan and response strategies to optimize the utilization of human resources and equipment [24]; address the health information needs based on the diverse socioeconomic, demographic, and ethnic factors [27]; re-design communication activities for a more effective dissemination of information related to the epidemic [28]; safeguarding the health of workforce [29] to ensure minimal impact on economy and involvement of the grassroot system and village health collaborators to combat pandemics [30, 31].

Thailand recorded the second lowest number of total cases (3,444) and deaths (58), and similarly the second lowest case rates (49) and death rates (0.8) per 1 million [26]. Surprisingly, we found that Thailand was the country with the highest IES-R and DASS-21 depression scores. This could be due to the impact of COVID-19 on the economy in Thailand. Among all MICs in Asia, the disruption on COVID-19 pandemic is the most severe on Thailand economy, due to its reliance on tourism as compared to other MICs. For 2020, the International Monetary Fund has predicted Thailand’s GDP to be reduced by 6.7 percent which is highest among Asian countries [32]. Pakistan ranked second in terms of DASS-21 scores and number of COVID cases and deaths. The congruence between psychological parameters and epidemiology of COVID-19 in Pakistan was due to poor sanitation, lack of basic preventive measures, lack of proper testing and medical facilities. Pakistani health professionals started protesting and threatened to quit work due to lack of Personal Protective Equipment (PPE) [33]. Currently, the vaccination coverage in rural Pakistan remains unsatisfactory amid various barriers including price, hesitancy, and low level of awareness [34]. Eid-ul-Adha is an annual religious festival that could not be cancelled due to religious obligations and led to a sharp spike in COVID-19 cases [35]. The unpreparedness and contradictory policies resulted in an alarming high rate of COVID-19 spread and worsening mental health and discrimination faced by Pakistani people. Iranian respondents demonstrated lowest confidence in their doctors. The economic sanctions that prevented medical supplies, equipment and drugs from arriving in Iran could lead to low confidence among Iranians [36].

This study highlighted unique protective factors for mental health in MICs of Asia. In this study, more than 90% of respondents agreed to wear masks to prevent COVID-19. During the initial stage of COVID-19 pandemic, medical and public health experts from the US and some European countries believed that there was no direct evidence of airborne transmission of COVID-19 [37]. In contrast, respiratory clinicians and public health experts from Asia argued that lack of evidence does not equate to evidence of ineffectiveness of face masks [38]. The use of face masks by Asians have played an important role in controlling the spread of COVID-19 [39]. This study showed the association between the use of face mask and lower DASS-21 anxiety and depression scores. This finding might support the postulation that wearing face mask could offer psychological benefits, such as feeling less vulnerable to infection via perceived control [37]. Staying with children and more than 6 people in the same household were protective factors due to the values of family support among Asians. Compared with western countries, family support has a greater influence on reducing the risk of adverse mental health in Asia [10].

The findings of this first multinational study have several implications for health and government policies. Firstly, the health authorities should offer psychological interventions to the general population who are at higher risk of developing adverse mental health including women, people younger than 30 years and single and separated status. High education background is a risk factor and online psychological interventions such as cognitive behaviour therapy (CBT) and mindfulness-based therapy could improve mental health for highly educated individual [40]. For countries with high IES-R scores (Thailand, China and Iran), online trauma-focused CBT that promotes trauma narration, problem solving related to problems associated with COVID-19 and home based relaxation could be helpful in reducing psychological impact [9]. Second, as physical symptoms resembling COVID-19 infection (e.g., rhinitis, persistent fever with cough, breathing difficulties) were associated with high IES-R and DASS-21 scores groups. There is an urgent need to develop accurate, rapid diagnostic tests in general practitioners’ clinics, community and rural settings [31]. A negative COVID-19 test result may alleviate anxiety, depression, stress and psychological impact. Enhancing the capacity of health system to combat COVID-19 may increase the confidence of public and improve mental health. Third, based on our findings, the WHO, governments and health authorities should provide regular updates on the effectiveness of vaccines and treatment methods. Mis-information related to the cause of COVID-19 [41], rumours [42] and inconsistent information [43] on COVID-19 symptoms, prevention, treatment and transmission mode were associated with negative psychological impact. Local governments, news agencies, professional and advocacy organisations should all provide health information and advices related to COVID-19 that are consistent with national guidelines and avoid mis-information [44]. It is important to identify group-specific demands would be helpful to provide proper information related to COVID-19 to fulfil the need of different population groups [27]. Various governments should offer relief packages to safeguard employment and economy to protect mental health. Additionally, the level of policy stringency in response to COVID-19 or pandemics, as measured by the Oxford Stringency Index, may influence mental health and should be moderated accordingly by respective governments [45].

This study has several limitations. First, the findings of this study were based on seven MICs in Asia and could not be generated to other countries. The study population had different sociodemographic characteristics as compared to the general population in the world due to sampling bias because only participants with Internet access could participate in this online survey. The respondent sampling method also compromised the representativeness of samples. The study population was female predominant (proportion of female in the study population: 67.76%; world population 49.58%) [46] and a high proportion of the study population possessed a university degree (85.6%). Thus, there is a potential risk of sampling bias because we could not reach out to potential respondents without Internet access. The second limitation was the cross-sectional nature of this study and inability to demonstrate cause and effect relationship. The third limitation was that we did not record demographic data regarding pre-existing mental illness of the study participants. The fourth limitation is that self-reported levels of psychological impact, anxiety, depression and stress may not always be aligned with objective assessment by mental health professionals. Nevertheless, psychological impact, anxiety, depression and stress are based on personal feelings, and self-reporting was paramount during the COVID-19 pandemic. The fifth limitation is that we did not study other aspects of the pandemic such as the potential threat of self-medication of hydroxychloroquine and cholorquine [47] and precautionary measures of walkthrough sanitization gates [48]. Lastly, we were unable to calculate the response rate. For potential respondents who were not keen to participate in the online survey, no response was recorded, and we could not collect any information from them.

## Conclusions

In conclusion, this multi-national study across 7 MICs in Asia showed that Thai reported the highest mean IES-R and DASS-21 anxiety, depression and stress scores. In contrast, Vietnamese reported the lowest mean scores in IES-R and DASS-21 anxiety, depression and stress scales. The risk factors for adverse mental health include age < 30 years, high education background, single and separated status, discrimination by other countries, contact with people with COVID-19 and worries about COVID-19. The protective factors for mental health include male gender, staying with children, staying with 6 or more people, employment, confidence in own’s doctors diagnosing COVID-19, high perceived likelihood of surviving COVID-19, spending less time on health information, hand hygiene practice and wearing a face mask.

## Supporting information

S1 Table Comparison of demographics of the participants from seven countries. (DOCX)

S2 Table Physical symptoms resembling COVID-19 infection reported by the participants from seven countries. (DOCX)

S3 Table Comparison of knowledge related to COVID-19 in participants of the seven countries. (DOCX)

S4 Table Comparison of precautionary measures related to COVID-19 in the participants of the seven countries. (DOCX)

S5 Table Comparison of information needs about COVID-19 in the participants of the seven Asian countries. (DOCX)
