# Supplementary material for: The impact of COVID-19 pandemic on physical and mental health of Asians: A study of seven middle-income countries in Asia
Source: PLoS One. 2021 Feb 11;16(2):e0246824. doi: 10.1371/journal.pone.0246824 (PMC7877638; doi:10.1371/journal.pone.0246824)
Supplement: S1 Table — (DOCX) [file pone.0246824.s001.docx]

**S1 Table.** Comparison of demographics of the participants from seven countries.

| Variable | | China  (N=1210) | | Philippines  (N=849) | | Iran  (N=550) | | Pakistan  (N=506) | | Vietnam  (N=122) | | Malaysia  (N=724) | | Thailand  (N=518) | | Total  (N=4479) | | $\chi^{2}$(*p*) |
| --- | --- | --- | --- | --- | --- | --- | --- | --- | --- | --- | --- | --- | --- | --- | --- | --- | --- | --- |
| ***Gender n* (%)** |  | |  | |  | |  | |  | |  | |  | |  | |  | |
| Male | | 396(32.7) | | 246(29.0) | | 149(27.1) | | 240(47.4) | | 45(36.9) | | 248(34.3) | | 120(23.2) | | 1444 | | 86.566  (*p*<0.001) |
| Female | | 814(67.3) | | 603(71.0) | | 401(72.9) | | 266(52.6) | | 77(63.1) | | 476(65.7) | | 398(76.8) | | 3035 | |  |
| ***Age n* (%)** | | | | | | | | | | | | | | | | | | |
| 12-21 | | 344(28.4) | | 230(27.1) | | 159(28.9) | | 87(17.2) | | 9(7.4) | | 78(10.8) | | 77(14.9) | | 984 | | 775.709  (*p*<0.001) |
| 22-30 | | 643(53.2) | | 226(26.6) | | 151(27.5) | | 170(33.6) | | 23(18.9) | | 230(31.8) | | 171(33.0) | | 1614 | |  |
| 31-40 | | 94(7.8) | | 178(21.0) | | 143(26.0) | | 132(26.1) | | 48(39.3) | | 99(13.7) | | 64(12.3) | | 758 | |  |
| 41-49 | | 90(7.4) | | 129(15.2) | | 65(11.8) | | 72(14.2) | | 21(17.2) | | 127(17.5) | | 75(14.5) | | 579 | |  |
| 50 and above | | 39(3.2) | | 86(10.1) | | 32(5.8) | | 45(8.9) | | 20(16.4) | | 190(26.2) | | 131(25.3) | | 543 | |  |
| ***Education Level n (%)*** | | | | | | | | | | | | | | | | | | |
| 1.Secondary level and below | | 65(5.4) | | 5(0.6) | | 25(4.5) | | 4(0.8) | | 1(0.8) | | 97(13.4) | | 7(1.4) | | 204 | | 444.444  (*p*<0.001) |
| 2. High School | | 81(6.7) | | 68(8.0) | | 0(0.0) | | 57(11.3) | | 13(10.7) | | 169(23.3) | | 53(10.2) | | 441 | |  |
| 3.Degree Holders | | 1064(87.9) | | 776(91.4) | | 525(95.5) | | 445(87.9) | | 108(88.5) | | 458(63.3) | | 458(88.4) | | 3834 | |  |
| ***Marital Status n (%)*** | | | | | | | | | | | | | | | | | | |
| Single | | 273(22.6) | | 585(68.9) | | 272(49.5) | | 244(48.2) | | 29(23.8) | | 318(43.9) | | 331(63.9) | | 2052 | | 677.661  (*p*<0.001) |
| Married | | 925(76.5) | | 240(28.3) | | 278(50.5) | | 236(46.6) | | 91(74.6) | | 385(53.2) | | 162(31.3) | | 2317 | |  |
| Divorced or Separated | | 9(0.7) | | 19(2.2) | | 0(0.0) | | 14(2.8) | | 1(0.8) | | 9(1.2) | | 19(3.7) | | 71 | |  |
| Widowed | | 3(0.2) | | 5(0.6) | | 0(0.0) | | 12(2.4) | | 1(0.8) | | 12(1.7) | | 6(1.1) | | 39 | |  |
| ***Parental Status n (%)*** | | | | | | | | | | | | | | | | | | |
| Has children | | 824(68.1) | | 241(28.4) | | 266(48.4) | | 212(41.9) | | 73(59.8) | | 329(45.4) | | 62(12.0) | | 2007 | | 599.680  (*p*<0.001) |
| No children | | 386(31.9) | | 608(71.6) | | 284(51.6) | | 294(58.1) | | 49(40.2) | | 395(54.6) | | 456(88.0) | | 2472 | |  |
| ***Family Size n (%)*** | | | | | | | | | | | | | | | | | | |
| 6 people or more | | 171(14.1) | | 276(32.5) | | 66(12.0) | | 212(41.9) | | 14(11.5) | | 276(38.1) | | 67(12.9) | | 1082 | | 565.282  (*p*<0.001) |
| 3-5 people | | 976(80.7) | | 453(53.4) | | 352(64.0) | | 250(49.4) | | 89(73.0) | | 386(53.3) | | 379(73.2) | | 2885 | |  |
| 2 people | | 52(4.3) | | 67(7.9) | | 55(10.0) | | 28(5.5) | | 14(11.5) | | 46(6.4) | | 49(9.5) | | 311 | |  |
| 1 person | | 11(0.9) | | 53(6.2) | | 77(14.0) | | 16(3.2) | | 5(4.0) | | 16(2.2) | | 23(4.4) | | 201 | |  |
| ***Employment Status n (%)*** | | | | | | | | | | | | | | | | | | |
| Unemployed | | 67(5.5) | | 31(3.7) | | 31(5.6) | | 31(6.1) | | 1(0.8) | | 50(6.9) | | 20(3.9) | | 231 | | 583.206  (*p*<0.001) |
| Housewife | | 24(2.0) | | 26(3.1) | | 42(7.6) | | 55(10.9) | | 1(0.8) | | 49(6.8) | | 23(4.4) | | 220 | |  |
| Retired | | 7(0.6) | | 10(1.1) | | 12(2.2) | | 8(1.6) | | 12(9.8) | | 37(5.1) | | 49(9.5) | | 135 | |  |
| Student | | 639(52.8) | | 296(34.9) | | 235(42.8) | | 137(27.1) | | 2(1.6) | | 219(30.2) | | 313(60.4) | | 1841 | |  |
| Employed | | 473(39.1) | | 486(57.2) | | 230(41.8) | | 275(54.3) | | 106(87.0) | | 369(51.0) | | 113(21.8) | | 2052 | |  |
