# Supplementary material for: The impact of COVID-19 pandemic on physical and mental health of Asians: A study of seven middle-income countries in Asia
Source: PLoS One. 2021 Feb 11;16(2):e0246824. doi: 10.1371/journal.pone.0246824 (PMC7877638; doi:10.1371/journal.pone.0246824)
Supplement: S2 Table — (DOCX) [file pone.0246824.s002.docx]

**S2 Table.** Physical symptoms resembling COVID-19 infection reported by the participants from seven countries.

| Variable | China  (N=1210) | Philippines  (N=849) | Iran  (N=550) | Pakistan  (N=506) | Vietnam  (N=122) | Malaysia  (N=724) | Thailand  (N=518) | | Total  (N=4479) | χ^2^(*p*) |
| --- | --- | --- | --- | --- | --- | --- | --- | --- | --- | --- |
| ***Prolonged or Recurring Fever n（%）*** | | | | | | | |  |  |  |
| Yes | 6(0.5) | 8(0.9) | 525(95.5) | 80(15.8) | 0(0.0) | 2(0.3) | 4(0.8) | | 625 | 3553.914  (*p*<0.001) |
| No | 1204(99.5) | 841(99.1) | 25(4.5) | 426(84.2) | 122(100) | 722(99.7) | 514(99.2) | | 3854 |  |
| ***Rigors or Chills n（%）*** | | | | | | | |  |  |  |
| Yes | 42(3.5) | 20(2.4) | 520(94.5) | 27(5.3) | 4(3.3) | 3(0.4) | 4(0.8) | | 620 | 3432.407  (*p*<0.001) |
| No | 1168(96.5) | 829(97.6) | 30(5.5) | 479(94.7) | 118(96.7) | 721(99.6) | 514(99.2) | | 3859 |  |
| ***Headache n（%）*** | | | | | | | |  |  |  |
| Yes | 117(9.7) | 212(25.0) | 367(66.7) | 183(36.2) | 60(49.2) | 40(5.5) | 57(11.0) | | 1036 | 976.853  (*p*<0.001) |
| No | 1093(90.3) | 637(75.0) | 183(33.3) | 323(63.8) | 62(50.8) | 684(94.5) | 461(89.0) | | 3443 |  |
| ***Myalgia n（%）*** | | | | | | | |  |  |  |
| Yes | 95(7.9) | 101(11.9) | 467(84.9) | 50(9.9) | 12(9.8) | 8(1.1) | 43(8.3) | | 776 | 2033.939  (*p*<0.001) |
| No | 1115(92.1) | 748(88.1) | 83(15.1) | 456(90.1) | 110(90.2) | 716(98.9) | 475(91.7) | | 3703 |  |
| ***Cough n（%）*** | | | | | | | |  |  |  |
| Yes | 168(13.9) | 128(15.1) | 441(80.2) | 142(28.1) | 28(23.0) | 22(3.0) | 50(9.7) | | 979 | 1370.084  (*p*<0.001) |
| No | 1042(86.1) | 721(84.9) | 109(19.8) | 364(71.9) | 94(77.0) | 702(97.0) | 468(90.3) | | 3500 |  |
| ***Breathing Difficulties n（%）*** | | | | | | | |  |  |  |
| Yes | 5(0.4) | 61(7.2) | 501(91.1) | 45(8.9) | 5(4.1) | 6(0.8) | 9(1.7) | | 632 | 3102.532  (*p*<0.001) |
| No | 1205(99.6) | 788(92.8) | 49(8.9) | 461(91.1) | 117(95.9) | 718(99.2) | 509(98.3) | | 3847 |  |
| ***Dizziness n（%）*** | | | | | | | |  |  |  |
| Yes | 88(7.3) | 48(5.7) | 493(89.6) | 43(8.5) | 22(18.0) | 14(1.9) | 25(4.8) | | 733 | 2485.287  (*p*<0.001) |
| No | 1122(92.7) | 801(94.3) | 57(10.4) | 463(91.5) | 100(82.0) | 710(98.1) | 493(95.2) | | 3746 |  |
| ***Rhinitis n (%)*** | | | | | | | |  |  |  |
| Yes | 205(16.9) | 112(13.2) | 470(85.5) | 11(2.2) | 33(27.0) | 0(0.0) | 14(2.7) | | 845 | 1968.235  (*p*<0.001) |
| No | 1005(83.1) | 737(86.8) | 80(14.5) | 495(97.8) | 89(73.0) | 724(100.0) | 504(97.3) | | 3634 |  |
| ***Sore Throat n（%）*** | | | | | | | |  |  |  |
| Yes | 139(11.5) | 108(12.7) | 446(81.1) | 89(17.6) | 23(18.9) | 13(1.8) | 46(8.9) | | 864 | 1599.428  (*p*<0.001) |
| No | 1071(88.5) | 741(87.3) | 104(18.9) | 417(82.4) | 99(81.1) | 711(98.2) | 472(91.1) | | 3615 |  |
| ***Recurrent Fever with Cough or Breathing Difficulties n（%）*** | | | | | | | |  |  |  |
| Yes | 4(0.3) | 1(0.1) | — | 45(8.9) | 0(0.00) | 1(0.1) | 4(0.8) | | 55 | 237.546  (*p*<0.001) |
| No | 1206(99.7) | 848(99.9) | — | 461(91.1) | 122(100) | 723(99.9) | 514(99.2) | | 3874 |  |
| ***Nausea, Vomiting or Diarrhea n（%）*** | | | | | | | | | | |
| Yes | — | 25(2.9) | 498(90.5) | 26(5.1) | 2(1.6) | 3(0.4) | 7(1.4) | | 561 | 2510.203  (p<0.001) |
| No | — | 824(97.1) | 52(9.5) | 480(94.9) | 120(98.4) | 721(99.6) | 511(98.6) | | 2708 |  |
| ***GP visit n（%）*** | | | | | | | |  |  |  |
| Yes | 42(3.5) | 35(4.1) | 53(9.6) | 139(27.5) | **6(4.9)** | 49(6.8) | 42(8.1) | | 366 | 306.728  (p<0.001) |
| No | 1168(96.5) | 814(95.9) | 497(90.4) | 367(72.5) | **88(72.1)** | 675(93.2) | 465(89.8) | | 4074 |  |
| ***Hospitalization n（%）*** | | | | | | | |  |  |  |
| Yes | 4(0.3) | 4(0.5) | **3(0.5)** | **83(16.4)** | **—** | 4(0.6) | 23(4.4) | | 121 | 601.817  (p<0.001) |
| No | 1206(99.7) | 845(99.5) | **50(9.1)** | **240(47.4)** | **94(77.0)** | 720(99.4) | 495(95.6) | | 3650 |  |
| ***Tested for COVID-19 n（%）*** | | | | | | | |  |  |  |
| Yes | 11(0.9) | 6(0.7) | **8(1.5)** | **87(17.2)** | **2(1.6)** | 49(6.8) | 12(2.3) | | 175 | 466.041 |
| No | 1199(99.1) | 843(99.3) | **45(8.2)** | **231(45.7)** | **92(75.4)** | 675(93.2) | 505(97.5) | | 3590 | (p<0.001) |
| ***Isolation n (%)*** | | | | | | | |  |  |  |
| Yes | 26(2.1) | 15(1.8) | **5(0.9)** | **90(17.8)** | **2(1.6)** | 43(5.9) | 11(2.1) | | 192 | 412.276 |
| No | 1184(97.9) | 834(98.2) | **48(8.7)** | **226(44.7)** | **92(75.4)** | 681(94.1) | 506(97.7) | | 3571 | (p<0.001) |
| ***Self Assessment for health status n (%)*** | | | | | | | |  |  |  |
| Not good or Very poor | 11(0.9) | 6(0.7) | 14(2.5) | 14(2.8) | **1(0.8)** | 3(0.4) | 11(2.1) | | 60 | 489.893  (p<0.001) |
| Normal | 372(30.7) | 130(15.3) | 138(25.1) | 61(12.1) | **21(17.2)** | 93(12.8) | 298(57.6) | | 1113 |  |
| Good or Very Good | 827(68.4) | 713(84.0) | 398(72.4) | 431(85.2) | **72(59.0)** | 628(86.8) | 209(40.3) | | 3278 |  |
| ***History of Chronic Disease n（%）*** | | | | | | | |  |  |  |
| Yes | 78(6.4) | 151(17.8) | 48(8.7) | **79(15.6)** | **13(10.7)** | 195(26.9) | 126(24.3) | | 690 | 201.232 |
| No | 1132(93.6) | 698(82.2) | 502(91.3) | **366(72.3)** | **81(66.4)** | 529(73.1) | 392(75.7) | | 3700 | (p<0.001) |
| ***Health Insurance n（%）*** | | | | | | | |  |  |  |
| Yes | 1118(92.4) | 468(55.1) | 56(10.2) | 161(31.8) | — | 427(59.0) | 340(65.6) | | 2570 | 1268.885 |
| No | 92(7.6) | 381(44.9) | 494(89.8) | 345(68.2) | — | 297(41.0) | 178(34.4) | | 1787 | (p<0.001) |
