# Supplementary material for: The impact of COVID-19 pandemic on physical and mental health of Asians: A study of seven middle-income countries in Asia
Source: PLoS One. 2021 Feb 11;16(2):e0246824. doi: 10.1371/journal.pone.0246824 (PMC7877638; doi:10.1371/journal.pone.0246824)
Supplement: S3 Table — (DOCX) [file pone.0246824.s003.docx]

**S3 Table.** Comparison of Knowledge related to COVID-19 in participants of the seven countries.

| Variable | China  (N=1210) | Philippines  (N=849) | Iran  (N=550) | Pakistan  (N=506) | Vietnam  (N=122) | Malaysia  (N=724) | | Thailand  (N=518) | Total  (N=4479) | χ^2^(*p*) |
| --- | --- | --- | --- | --- | --- | --- | --- | --- | --- | --- |
| ***Mode of transmission n (%)*** | | | | | | |  |  |  |  |
| ***Droplets n (%)*** | | | | | | |  |  |  |  |
| Agree | 1115(92.1) | 839(98.8) | 390(70.9) | 408(80.6) | 94(77.0) | 657(90.7) | | 513(99.0) | 4016 | 409.082  (*p*<0.001) |
| Disagree or uncertain | 95(7.9) | 10(1.2) | 160(29.1) | 98(19.4) | 28(23.0) | 67(9.3) | | 5(1.0) | 463 |  |
| ***Contact with Contaminated Surfaces n (%)*** | | | | | | |  |  |  |  |
| Agree | 892(73.7) | 822(96.8) | 535(97.3) | 423(83.6) | 122(100) | 414(57.2) | | 500(96.5) | 3708 | 695.403  (*p*<0.001) |
| Disagree or uncertain | 318(26.3) | 27(3.2) | 15(2.7) | 83(16.4) | 0(0.0) | 310(42.8) | | 18(3.5) | 771 |  |
| ***Airborne n (%)*** | | | | | | |  |  |  |  |
| Agree | 732(60.5) | 289(34.0) | 292(53.1) | 213(42.1) | 73(59.8) | 250(34.5) | | 65(12.5) | 1914 | 433.766  (*p*<0.001） |
| Disagree or uncertain | 478(39.5) | 560(66.0) | 258(46.9) | 293(57.9) | 49(40.2) | 474(65.5) | | 453(87.5) | 2565 |  |
| ***Level of Confidence in one’s own doctor in diagnosing or recognizing COVID-19 n (%)*** | | | | | | |  |  |  |  |
| 1. Very confident | 563(46.5) | 231(27.2) | 101(18.4) | 125(24.7) | 47(38.5) | 407(56.2) | | 114(22.0) | 1588 | 738.642  (*p*<0.001) |
| 2. Confident | 561(46.4) | 422(49.7) | 259(47.1) | 192(37.9) | 56(45.9) | 272(37.6) | | 292(56.4) | 2054 |  |
| 3. Not confident | 50(4.1) | 118(13.9) | 58(10.5) | 105(20.8) | 10(8.2) | 21(2.9) | | 98(18.9) | 460 |  |
| 4. Very unconfident | 36(3.0) | 78(9.2) | 132(24.0) | 84(16.6) | 9(7.4) | 24(3.3) | | 14(2.7) | 377 |  |
| ***Likelihood of Contracting COVID-19 during the pandemic n (%)*** | | | | | | |  |  |  |  |
| 1. Very possible | 135(11.2) | 66(7.8) | 33(6.0) | 84(16.6) | 9(7.4) | 143(19.8) | | 107(20.7) | 577 | 982.778  (*p*<0.001) |
| 2. Somewhat possible | 358(29.6) | 270(31.8) | 207(37.6) | 188(37.2) | 15(12.3) | 384(53.0) | | 252(48.6) | 1674 |  |
| 3. Not very possible | 437(36.1) | 315(37.1) | 106(19.3) | 119(23.5) | — | 115(15.9) | | 114(22.0) | 1206 |  |
| 4. Impossible | 121(10.0) | 137(16.1) | 40(7.3) | 31(6.1) | 5(4.1) | 20(2.7) | | 28(5.4) | 382 |  |
| 5. Uncertain | 159(13.1) | 61(7.2) | 164(29.8) | 84(16.6) | 93(76.2) | 62(8.6) | | 17(3.3) | 640 |  |
| ***Likelihood of survival after contracting COVID-19 n (%)*** | | | | | | |  |  |  |  |
| 1. Very possible | 278(23.0) | 320(37.7) | 167(30.4) | 148(29.2) | 29(23.8) | 207(28.6) | | 175(33.8) | 1324 | 459.627  (*p*<0.001) |
| 2. Somewhat possible | 559(46.2) | 387(45.6) | 192(34.9) | 164(32.4) | 62(50.8) | 430(59.4) | | 290(56.0) | 2084 |  |
| 3. Not very possible | 124(10.2) | 64(7.5) | 20(3.6) | 52(10.3) | — | 24(3.3) | | 31(6.0) | 315 |  |
| 4. Impossible | 20(1.7) | 17(2.0) | 15(2.7) | 26(5.1) | 13(10.6) | 3(0.4) | | 11(2.1) | 105 |  |
| 5. Uncertain | 229(18.9) | 61(7.2) | 156(28.4) | 115(22.7) | 18(14.8) | 60(8.3) | | 11(2.1) | 650 |  |
| ***Level of satisfaction with the amount of health information available regarding COVID-19 n (%)*** | | | | | | |  |  |  |  |
| 1. Very satisfied | 485(40.1) | 125(14.7) | 78(14.2) | 109(21.5) | 63(51.6) | 358(49.4) | | 87(16.8) | 1305 | 699.925  (*p*<0.001) |
| 2. Somewhat satisfied | 423(35.0) | 518(61.0) | 289(52.5) | 279(55.2) | 56(45.9) | 345(47.7) | | 298(57.5) | 2208 |  |
| 3. Not satisfied | 211(17.4) | 169(19.9) | 78(14.2) | 74(14.6) | — | 11(1.5) | | 105(20.3) | 648 |  |
| 4. Very unsatisfied or Uncertain | 91(7.5) | 37(4.4) | 105(19.1) | 44(8.7) | 3(2.5) | 10(1.4) | | 28(5.4) | 318 |  |
| ***Level of worry about family members being diagnosed with COVID-19 n (%)*** | | | | | | |  |  |  |  |
| 1.Very worried | 417(34.5) | 512(60.4) | 236(43.0) | 316(62.5) | 41(33.6) | 497(68.6) | | 17(3.3) | 2036 | 1800.751  （*p*<0.001） |
| 2. Somewhat worried | 492(40.7) | 289(34.0) | 218(39.6) | 126(24.9) | 51(41.8) | 187(25.8) | | 67(12.9) | 1430 |  |
| 3. Not worried | 221(18.3) | 29(3.4) | 53(9.6) | 29(5.7) | 21(17.2) | 31(4.3) | | 238(45.9) | 622 |  |
| 4. Not worried at all | 70(5.8) | 5(0.6) | 40(7.3) | 14(2.7) | — | 5(0.7) | | 178(34.4) | 312 |  |
| 5. No family members | 10(0.7) | 14(1.6) | 3(0.5) | 21(4.2) | 9(7.4) | 4(0.6) | | 18(3.5) | 79 |  |
| ***Did you feel discriminated against by other countries after the outbreak n (%)*** | | | | | | |  |  |  |  |
| Yes | — | 146(17.2) | — | 216(42.7) | 39(32.0) | — | | 173(33.4) | 574 | 109.336  （*p*<0.001） |
| No | — | 703(82.8) | — | 290(57.3) | 83(68.0) | — | | 345(66.6) | 1421 |  |
| ***How much time did you spend monitoring information regarding the pandemic n (%)*** | | | | | | |  |  |  |  |
| 0-1 hours | — | 178(21.0) | — | 162(32.0) | 44(36.1) | — | | — | 384 | 65.210  （*p*<0.001） |
| 1-2 hours | — | 270(31.8) | — | 108(21.3) | 50(41.0) | — | | — | 428 |  |
| 2 or more hours | — | 401(47.2) | — | 236(46.6) | 19(15.6) | — | | — | 656 |  |
| ***Direct contact with people infected by COVID-19 n (%)*** | | | | | | | | | | |
| Yes | 4(0.3) | 21(2.5) | 8(1.5) | 70(13.8) | 0(0.0) | 5(0.7) | | 3(0.6) | 111 | 315.865  (*p*<0.001) |
| No | 1206(99.7) | 828(97.5) | 542(98.5) | 436(86.2) | 122(100) | 719(99.3) | | 515(99.4) | 4368 |  |
| ***Indirect Contact with people infected by COVID-19 n (%)*** | | | | | | | | | | |
| Yes | 6(0.5) | 41(4.8) | 15(2.7) | 68(13.4) | 8(6.6) | 8(1.1) | | 8(1.5) | 154 | 210.803  (*p*<0.001) |
| No | 1204(99.5) | 808(95.2) | 535(97.3) | 438(86.6) | 114(93.4) | 716(98.9) | | 510(98.5) | 4325 |  |
| ***Contact with materials contaminated with COVID-19 n (%)*** | | | | | | | | | | |
| Yes | 12(1.0) | 11(1.3) | 6(1.1) | 66(13.0) | 2(1.6) | 0(0.0) | | 32(6.2) | 129 | 258.771  (*p*<0.001) |
| No | 1198(99.0) | 838(98.7) | 544(98.9) | 439(86.8) | 117(95.9) | 724(100.0) | | 486(93.8) | 4346 |  |
