# Supplementary material for: The impact of COVID-19 pandemic on physical and mental health of Asians: A study of seven middle-income countries in Asia
Source: PLoS One. 2021 Feb 11;16(2):e0246824. doi: 10.1371/journal.pone.0246824 (PMC7877638; doi:10.1371/journal.pone.0246824)
Supplement: S4 Table — (DOCX) [file pone.0246824.s004.docx]

**S4 Table.** Comparison of Precautionary Measures related to COVID-19 in the Participants of the Seven Countries.

| Variable | China  (N=1210) | Philippines  (N=849) | Iran  (N=550) | Pakistan  (N=506) | Vietnam  (N=122) | [Malaysia](about:blank)  (N=724) | Thailand  (N=518) | | Total  (N=4479) | χ^2^(*p*) | |
| --- | --- | --- | --- | --- | --- | --- | --- | --- | --- | --- | --- |
| ***Covering your mouth when coughing or sneezing n (%)*** | | | | | | | |  |  |  |  |
| Yes | 1159(95.8) | 846(99.6) | 536(97.5) | 489(96.6) | 122(100) | 723(99.9) | 514(99.2) | | 4389 | 66.609 |  |
| No | 51(4.2) | 3(0.4) | 14(2.5) | 17(3.4) | 0(0.0) | 1(0.1) | 4(0.8) | | 90 | (*p*<0.001) |  |
| ***Avoidance of sharing cutlery during meals n (%)*** | | | | | | | |  |  |  |  |
| Yes | 1015(83.9) | 763(89.9) | 511(92.9) | 455(89.9) | 120(98.4) | 685(94.6) | 516(99.6) | | 4065 | 142.066 | |
| No | 195(16.1) | 86(10.1) | 39(7.1) | 51(10.1) | 2(1.6) | 39(5.4) | 2(0.4) | | 414 | (*p*<0.001) | |
| ***Washing your hands using soap or hand sanitizer n (%)*** | | | | | | | |  |  |  |  |
| Yes | 1177(97.3) | 849(100) | 543(98.7) | 498(98.4) | 122(100) | 724(100.0) | 516(99.6) | | 4429 | 30.285 | |
| No | 33(2.7) | 0(0.0) | 7(1.3) | 8(1.6) | 0(0.0) | 0(0.0) | 2(0.4) | | 50 | (*p*<0.001) | |
| ***Washing hands after coughing, sneezing or rubbing your nose n (%)*** | | | | | | | |  |  |  |  |
| Yes | 1135(93.8) | 838(98.7) | 520(94.5) | 468(92.5) | 122(100) | 717(99.0) | 509(98.3) | | 4309 | 113.852 | |
| No | 75(6.2) | 11(1.3) | 30(5.5) | 38(7.5) | 0(0.0) | 7(1.0) | 9(1.7) | | 170 | (*p*<0.001) | |
| ***Wearing a mask n (%)*** | | | | | | | |  |  |  |  |
| Yes | 1171(96.8) | 786(92.6) | 459(83.5) | 423(83.6) | 122(100) | 716(98.9) | 509(98.3) | | 4186 | 256.484 | |
| No | 39(3.2) | 63(7.4) | 91(16.5) | 83(16.4) | 0(0.0) | 8(1.1) | 9(1.7) | | 293 | *(p*<0.001) | |
| ***Washing hands after touching contaminated surfaces n (%)*** | | | | | | | |  |  |  |  |
| Yes | 1192(98.5) | 845(99.5) | 540(98.2) | 481(95.1) | 121(99.2) | 723(99.9) | 516(99.6) | | 4418 | 66.262 | |
| No | 18(1.5) | 4(0.5) | 10(1.8) | 25(4.9) | 1(0.8) | 1(0.1) | 2(0.4) | | 61 | (*p*<0.001) | |
| ***People are too worried about COVID-19 n (%)*** | | | | | | | |  |  |  |  |
| Yes | 672(55.5) | 650(76.6) | 61(11.1) | 438(86.6) | 89(73.0) | 655(90.5) | 469(90.5) | | 3034 | 1298.255 | |
| No | 538(44.5) | 199(23.4) | 489(88.9) | 68(13.4) | 33(27.0) | 69(9.5) | 49(9.5) | | 1445 | (*p*<0.001) | |
| ***Time spent at home n (%)*** | | | | | | | |  |  |  |  |
| 1.[0-10] | 39(3.2) | 220(25.9) | 38(6.9) | 118(23.3) | 94(77.0) | — | 128(24.7) | | 637 | 879.608  (*p*<0.001) | |
| 2.(10-20] | 146(12.1) | 162(19.1) | 108(19.6) | 122(24.1) | 14(11.5) | — | 201(38.8) | | 753 |  |  |
| 3.(20-24] | 1025(84.7) | 467(55.0) | 404(73.5) | 266(52.6) | 10(8.2) | — | 189(36.5) | | 2361 |  |  |
