# Supplementary material for: The impact of COVID-19 pandemic on physical and mental health of Asians: A study of seven middle-income countries in Asia
Source: PLoS One. 2021 Feb 11;16(2):e0246824. doi: 10.1371/journal.pone.0246824 (PMC7877638; doi:10.1371/journal.pone.0246824)
Supplement: S5 Table — (DOCX) [file pone.0246824.s005.docx]

**S5 Table**. Comparison of Information Needs about COVID-19 in the Participants of the Seven Asian Countries.

| Variable | China  (N=1210) | Philippines  (N=849) | Iran  (N=550) | Pakistan  (N=506) | Vietnam  (N=122) | [Malaysia](about:blank)  (N=724) | Thailand  (N=518) | | Total  (N=4479) | $\chi^{2}$(*p*) |  |
| --- | --- | --- | --- | --- | --- | --- | --- | --- | --- | --- | --- |
| ***Understanding of symptoms related to COVID-19 n (%)*** | | | | | | | |  |  |  |  |
| Yes | 1108(91.6) | 581(68.4) | 469(85.3) | 267(52.8) | 86(70.5) | 563(77.8) | 389(75.1) | | 3463 | 376.883  (*p*<0.001) |  |
| No | 102(8.4) | 268(31.6) | 81(14.7) | 239(47.2) | 36(29.5) | 161(22.2) | 129(24.9) | | 1016 |  |  |
| ***Prevention advice n (%)*** | | | | | | | |  |  |  |  |
| Yes | 1134(93.7) | 582(68.6) | 451(82.0) | 314(62.1) | 81(66.4) | 547(75.6) | 392(75.7) | | 3501 | 313.851  (p<0.001) |  |
| No | 76(6.3) | 267(31.4) | 99(18.0) | 192(37.9) | 41(33.6) | 177(24.4) | 126(24.3) | | 978 |  |  |
| ***Treatment advice n (%)*** | | | | | | | |  |  |  |  |
| Yes | 1000(82.6) | 606(71.4) | 497(90.4) | 333(65.8) | 68(55.7) | 594(82.0) | 396(76.4) | | 3494 | 172.626  (*p*<0.001) |  |
| No | 210(17.4) | 243(28.6) | 53(9.6) | 173(34.2) | 54(44.3) | 130(18.0) | 122(23.6) | | 985 |  |  |
| ***Need for regular information updates n (%)*** | | | | | | | |  |  |  |  |
| Yes | 1122(92.7) | 601(70.8) | 476(86.5) | 287(56.7) | 95(77.9) | 668(92.3) | 407(78.6) | | 3656 | 443.069  (*p*<0.001) |  |
| No | 88(7.3) | 248(29.2) | 74(13.5) | 219(43.3) | 27(22.1) | 56(7.7) | 111(21.4) | | 823 |  |  |
| ***Understanding of local outbreaks n (%)*** | | | | | | | |  |  |  |  |
| Yes | 1133(93.6) | 590(69.5) | 471(85.6) | 268(53.0) | 78(63.9) | 682(94.2) | — | | 3222 | 573.412  (*p*<0.001) |  |
| No | 77(6.4) | 259(30.5) | 79(14.4) | 236(46.6) | 44(36.1) | 42(5.8) | — | | 737 |  |  |
| ***Need for more personalized information, such as advice for those with pre-existing medical conditions n (%)*** | | | | | | | |  |  |  |  |
| Yes | 1171(96.8) | 577(68.0) | 453(82.4) | 276(54.5) | 77(63.1) | 605(83.6) | 386(74.5) | | 3545 | 594.855  (*p*<0.001) |  |
| No | 39(3.2) | 272(32.0) | 97(17.6) | 226(44.7) | 45(36.9) | 119(16.4) | 132(25.5) | | 930 |  |  |
| ***Understanding of effectiveness of drugs or vaccines available n (%)*** | | | | | | | |  |  |  |  |
| Yes | 1139(94.1) | 611(72.0) | 514(93.5) | 331(65.4) | 77(63.1) | 676(93.4) | 409(79.0) | | 3757 | 436.457  (*p*<0.001) |  |
| No | 71(5.9) | 238(28.0) | 36(6.5) | 170(33.6) | 45(36.9) | 48(6.6) | 109(21.0) | | 717 |  |  |
| **Understanding of number of infections and location n (%)** | | | | | | | |  |  |  |  |
| Yes | 1160(95.9) | 586(69.0) | 472(85.8) | 249(49.2) | 77(63.1) | 669(92.4) | 408(78.8) | | 3621 | 666.584  (*p*<0.001) |  |
| No | 50(4.1) | 263(31.0) | 78(14.2) | 252(49.8) | 45(36.9) | 55(7.6) | 110(21.2) | | 853 |  |  |
| ***Travel advice n (%)*** | | | | | | | |  |  |  |  |
| Yes | 1172(96.9) | 517(60.9) | 397(72.2) | 228(45.1) | 45(36.9) | 619(85.5) | 391(75.5) | | 3369 | 779.535  (*p*<0.001) |  |
| No | 38(3.1) | 332(39.1) | 153(27.8) | 275(54.3) | 77(63.1) | 105(14.5) | 127(24.5) | | 1107 |  |  |
| ***Understanding of modes of transmission of COVID-19 n (%)*** | | | | | | | |  |  |  |  |
| Yes | 1144(94.5) | 566(66.7) | 483(87.8) | 277(54.7) | 78(63.9) | 635(87.7) | 404(78.0) | | 3587 | 526.606  (*p*<0.001) |  |
| No | 66(5.5) | 283(33.3) | 67(12.2) | 229(45.3) | 44(36.1) | 89(12.3) | 114(22.0) | | 892 |  |  |
| ***Understanding of what methods and strategies are being utilized by other countries n (%)*** | | | |  |  |  |  | |  |  |  |
| Yes | 594(49.1) | 587(69.1) | 461(83.8) | 281(55.5) | 73(59.8) | 616(85.1) | 393(75.9) | | 3005 | 406.623  (p<0.001) |  |
| No | 616(50.9) | 262(30.9) | 89(16.2) | 225(44.5) | 49(40.2) | 108(14.9) | 125(24.1) | | 1474 |  |  |
